# Effect of N-acetylcysteine on hair follicle changes in mouse model of cyclophosphamide-induced alopecia: histological and biochemical study

**DOI:** 10.1007/s00418-024-02282-0

**Published:** 2024-04-20

**Authors:** Yomna F. Hassan, Dalia A. Shabaan

**Affiliations:** Medical Histology and Cell Biology Department, Faculty of Medicine, Mansoura University, Mansoura, Egypt

**Keywords:** Cyclophosphamide, N-acetylcysteine, Hair follicle, p53, ki67, Oxidative stress

## Abstract

Chemotherapy-induced alopecia (CIA) represents one of the most severe side effects of chemotherapy, which forces some patients to reject cancer treatment. The exact pathophysiological mechanisms of CIA are not clearly understood, which makes it difficult to discover efficient preventive or therapeutic procedures for this adverse effect. N-acetylcysteine (NAC) has a strong antioxidant activity as it stimulates glutathione synthesis and acts as an oxygen radical scavenger. The current study tried to investigate the efficacy of NAC in preserving biochemical parameters and hair follicle structure against cyclophosphamide (CYP) administration. In total, 40 adult female C57BL/6 mice were induced to enter anagen by depilation (day 0) and divided into four groups: group I (control), group II (CYP) received a single dose of CYP [150 mg/kg body weight (B.W.)/intraperitoneal injection (IP)] at day 9, group III (CYP & NAC) received a single dose of CYP at day 9 as well as NAC (500 mg/kg B.W./day/IP) from day 6–16, and group IV (NAC) received NAC from day 6–16. CYP administration in group II induced an increase in malondialdehyde (MDA), decrease in superoxide dismutase (SOD), histological hair follicle dystrophy, disruption of follicular melanogenesis, overexpression of p53, and loss of ki67 immunoreactivity. NAC coadministration in group III reversed CYP-induced alterations in the biochemical parameters and preserved hair follicle structure, typical follicular melanin distribution as well as normal pattern of p53 and ki67 expression. These findings indicated that NAC could be used as an efficient and safe therapeutic option for hair loss induced by chemotherapy.

## Introduction

Hair follicles (HFs) are essential thin skin components responsible for hair generation. They undergo cyclic stages, consisting of the anagen (growth phase), catagen (transition phase), and telogen (resting phase) (Kim et al. 2021). The anagen HF is formed of four main anatomic regions: infundibulum, isthmus, suprabulbar region, and hair bulb (Welle 2023). The infundibulum extends from the opening of the HF in the epidermis to the opening of the sebaceous gland duct. The isthmus includes the part of the HF spreading from the entrance of the sebaceous gland duct to approximately the attachment of the arrector pili muscle. The suprabulbar region is the area between the insertion of the arrector pili muscle to the cornified part of the bulb, then, the bulb stretches from the end of the cornified part to the base of the HF (Pinedo-Moraleda et al. 2023). The infundibulum and the isthmus represent the upper permanent portion of the HF, whereas the suprabulbar region and the hair bulb constitute the inferior transitory segment which undergoes regression during catagen and is absent during telogen (Wiener 2021).

The majority of anticancer agents cause disruption of the HF structure, resulting in serious massive hair loss (alopecia) (Kim et al. 2021). Rapidly proliferating anagen HFs and their pigmentary system are the main targets of chemotherapy-induced HF damage (Yoon et al. 2016). Chemotherapy-induced alopecia (CIA) represents one of the most severe side effects of chemotherapy, which forces some patients to reject treatment, especially women. CIA is usually reversible, and hair regrowth occurs within 3–6 months after termination of chemotherapy. However, the possibility of abnormally structured or discolored new hair as well as the risk of permanent CIA have been increasingly reported (Santos et al. 2021; Piccini et al. 2022).

Cyclophosphamide (CYP) is a broad-spectrum alkylating agent used in the treatment of different types of cancers, multiple sclerosis, and systemic lupus erythematosus (Zhang et al. 2022). CYP is widely used in clinical practice because of its high curative effect; however, it damages not only cancerous cells but also other rapidly proliferating normal cells in the body (Ou et al. 2021). After systemic administration, CYP is converted to its major active metabolite 4-hydroxycyclophosphamide in the liver by the cytochrome-P450 enzyme system. CYP active metabolites form DNA cross-links and produce more reactive oxygen species (ROS) (Huang et al. 2021).

The ROS are counteracted naturally by antioxidant defense systems, such as superoxide dismutase (SOD), which is considered an essential antioxidant marker present in almost all cells to provide the first line of defense against the toxic effects of ROS accumulation by converting them into less reactive hydrogen peroxide. However, CYP administration is usually accompanied by the generation of excessive free radicals and ROS, causing a depletion of antioxidant enzymes, and eventually an increase in lipid peroxidation and induction of oxidative damage (Zhao et al. 2023). Malondialdehyde (MDA) is one of the most important products of lipid peroxidation and a useful biomarker for oxidative stress (Cordiano et al. 2023). Therefore, serum levels of SOD and MDA are commonly utilized as oxidative stress indicators to assess the patients’ therapeutic response to chemotherapy (Lu et al. 2023).

CYP-induced oxidative stress causes cell death and apoptosis in the rapidly-dividing anagen HFs leading to disturbance in the proliferation of matrix keratinocytes in the bulb region, loss of hair integrity, breakage and falling-off of the hair resulting in either transient or permanent alopecia (Huang et al. 2021). CYP‐induced alopecia in mice is a well-established animal model of CIA, and the possible mechanisms underlying the response of the HFs to CYP treatment have been shown (Chen et al. 2016; Onaolapo et al. 2018; Yoneda et al. 2021). Aiba et al. (2023) have reported that CYP is selected as the anticancer agent in CIA studies because it is widely used especially in the treatment of breast cancer and it causes high rates of CIA in about 96% of breast cancer patients.

A number of procedures and reagents have been used to protect against CIA, such as scalp tourniquets, scalp cooling, and pharmacological agents. Despite significant advances in research, no effective and safe approach has been proved for this severe, psychologically distressing adverse effect in oncologic patients (Wikramanayake et al. 2023). Therefore, the development of more satisfactory management strategies for CIA remains a major research challenge.

Antioxidants have been recently shown to possess ability in alleviating toxicity resulting from chemotherapy. Besides improving the therapeutic efficiency of chemotherapeutic agents, antioxidants prevent cell damage by eliminating free radicals and other ROS produced by chemotherapy (Yoneda et al. 2021). N-acetylcysteine (NAC), the acetylated derivative of the amino acid L-cysteine, was originally used as a mucolytic agent to alleviate symptoms of cystic fibrosis. Currently, NAC is effective in the treatment of paracetamol overdose and acute heavy metal poisoning (Mlejnek 2022). NAC has a strong antioxidant activity as it stimulates glutathione (GSH) synthesis and acts as an oxygen radical scavenger (Tieu et al. 2023).

NAC has been shown to prevent CYP-induced alopecia in a newborn rat model (Jimenez et al. 1992; Hussein and Ardalan 1993) and doxorubicin-induced alopecia in an adult mouse model (D’Agostini et al. 1998). However, these studies did not illustrate the microscopic changes in the HFs and their different segments, the oxidative stress parameters or the exact mechanism of NAC action and its effect on HF apoptosis and proliferation. Moreover, to our knowledge, the applicability of NAC in the clinical trials to prevent CIA has not yet been reported. Therefore, the current study tried to investigate and highlight the efficacy of NAC in preserving HF structure and biochemical parameters against CYP using depilated adult C57BL/6 mice model.

In C57BL/6 mice, hair shafts are well pigmented and melanin-producing melanocytes are present only in the HFs, with no other melanocytes located in the epidermis. Melanin production is associated with the anagen phase of hair growth. Thus, such combination of follicular melanogenesis and follicular cyclic changes leads to characteristic changes in skin pigmentation during anagen development. Therefore, C57BL/6 mice are the most commonly used model for hair growth studies (Chung et al. 2017).

## Materials and methods

### Chemicals

CYP was purchased from Baxter Oncology GmbH (Halle, Germany) in the form of Endoxan vials (1 g dry powder/vial). NAC was purchased from Zambon (Vicenza, Italy) in the form of Fluimucil ampoules (300 mg/3 ml ampoule).

### Experimental animals

In total, 40 6-week-old adult female C57BL/6 mice were used in this study. The animals were obtained from Mansoura Experimental Research Center (MERC), Mansoura University, Egypt. Mice were kept in plastic cages under adequate ventilation and temperature and exposed to a 12 h light/dark cycle 2 weeks before the experiment for adaptation and fed a standard laboratory diet and water ad libitum.

### Induction of anagen

As most of the human scalp HFs remain in anagen, animal models clinically relevant to CIA model should be aligned with anagen, corresponding to the patient’s CIA (Yoneda et al. 2021). To synchronize the anagen phase of the hair growth cycle, the mice were induced to enter anagen by depilation. A 6 cm^2^ area (longitudinal length: 3 cm & horizontal length: 2 cm) of the black fur of the dorsal skin in all mice was depilated on day 0 (Chen et al. 2016). By this technique, all depilated HFs directly began to transform into the same stage of anagen development. Progressive changes were typically seen in dorsal skin pigmentation of all mice which developed mature anagen-VI stage follicles within 9–10 days, followed by the appearance of new hair shafts (Kim et al. 2022). The hair changes on the back of the mice were observed daily and photographed on days 0, 9, 12, and 16 after depilation.

### Animal grouping

Mice were equally divided into four main groups. Group I (control group) received normal saline at a dose of 0.5 ml/day by intraperitoneal injection throughout the experimental period (from day 0 to day 16). Group II (CYP group) received CYP in a single intraperitoneal dose of 150 mg/kg body weight (Kim et al. 2021) dissolved in saline at day 9. Group III (CYP & NAC group) received CYP in the same single intraperitoneal dose of 150 mg/kg body weight as group II (Kim et al. 2021) at day 9 as well as NAC in a dose of 500 mg/kg body weight/day intraperitoneally (Abdelrahman et al. 2010) starting from day 6 till the end of the experiment (from day 6 to day 16). Group IV (NAC group) received NAC in a dose of 500 mg/kg body weight/day intraperitoneally (Abdelrahman et al. 2010) starting from day 6 till the end of the experiment (from day 6 to day 16).

### Obtaining the specimens

At the end of the experiment (on day 16 after depilation), mice were anesthetized by intraperitoneal injection of 70 mg/kg body weight thiopental sodium (Sakena et al. 2020), and blood samples were collected from the left ventricle of the heart for biochemical study. Then, the animals were perfused through the left ventricle with 500 ml 10% neutral buffered formalin. Back skin from the depilated site was dissected out from all animals to be prepared for histological study.

### Biochemical study

For assessment of MDA, serum was mixed with trichloroacetic acid (TCA, 15% w/v), thiobarbituric acid (TBA, 0.375% w/v), and hydrochloric acid (HCl 0.25 N). The solution was placed in a boiling water bath for 15 min, then, cooled and centrifuged at 1000 g for 10 min. The absorbance of the supernatant was determined using a spectrophotometer (Momeni and Eskandari 2020). To assess SOD, serum was mixed with 2 ml of a solution of 0.1 mM EDTA, 50 mM sodium carbonate and 96 mM nitro blue tetrazolium (NBT) containing 0.05 ml hydroxylamine. Then, the auto-oxidation of hydroxylamine was measured spectrophotometrically (Panda et al. 2018).

### Histological study

The skin was postfixed in 10% neutral buffered formalin for 24 h and processed for light microscopic study. Paraffin sections (4–5 μm) were prepared and stained with hematoxylin and eosin (H&E) stain (Bancroft and Layton 2019) for routine histological examination, Schmorl’s stain (Orchard 2019) for demonstration of melanin and immunohistochemical stain (Magaki et al. 2019) with anti-p53 antibody (class immunoglobulin G, clone EP9, Biocare Medical, United States) and anti-ki67 antibody (class immunoglobulin G, clone SP6, Master Diagnóstica, Spain) for demonstration of apoptosis and proliferation, respectively. Then, slides were photographed using an Olympus® digital camera (SC100) installed on an Olympus^®^ light microscope (CX31; Japan).

### Immunohistochemical staining

After deparaffinization and rehydration of the slides, they were incubated in hydrogen peroxide for 15 min to block endogenous peroxidase activity, then washed in phosphate-buffered saline (PBS) at pH 7.4 for 10 min. Sections were heated in citrate buffer (pH 6.0) for 10 min for antigen retrieval and incubated in 1% bovine serum albumin (BSA) dissolved in PBS at 37 °C for 20 min to prevent nonspecific background staining. After that, the slides were incubated with the primary antibodies, anti-p53 and anti-ki67 (rabbit monoclonal antibodies, dilution 1:50–1:100), at room temperature for 60 min, washed, and incubated with secondary antibodies (biotinylated goat antipolyvalent) in a humidity chamber for 10 min. Then, the sections were incubated with streptavidin peroxidase and the reaction was visualized by adding 3,3α-diaminobenzidene tetrahydrochloride (DAB) to the sections. Finally, slides were counterstained with Mayer’s hematoxylin, dehydrated, cleared in xylene, and coverslipped. After omitting the primary antibodies, negative control skin sections were placed under the same conditions. Additionally, specimens from colon carcinoma and tonsil (Küçükosmanoğlu et al. 2022) were used as positive controls to confirm the specificities of anti-p53 and anti-ki67 antibodies, respectively.

### Morphometric study

The resultant images of anti-p53 and anti-ki65 stained slides were analyzed on an Intel® Core I3® based computer using Video Test Morphology® software (Video Test, Saint Petersburg, Russia) with a specific built-in routine for calibrated distance measurement, area measurement, automated object analysis, and color intensity. It was used to assess the mean percentage area of p53 and ki67/high power field (HPF; × 400) in the bulb region of twenty randomly chosen HFs from each animal in all groups.

### Statistical analysis

The biochemical and morphometric data were tabulated, coded and then analyzed using the computer program statistical package for social science (SPSS) version 17.0. Comparisons between groups were carried out using ANOVA (analysis of variance) to compare between more than two groups of numerical (parametric) data followed by post-hoc Tukey for multiple comparisons. A *P* (probability) value < 0.05 was considered statistically significant, while *P* value < 0.001 was considered highly significant (Hazra and Gogtay 2016).

## Results

### Macroscopic results (photodocumentation)

**Fig. 1:**
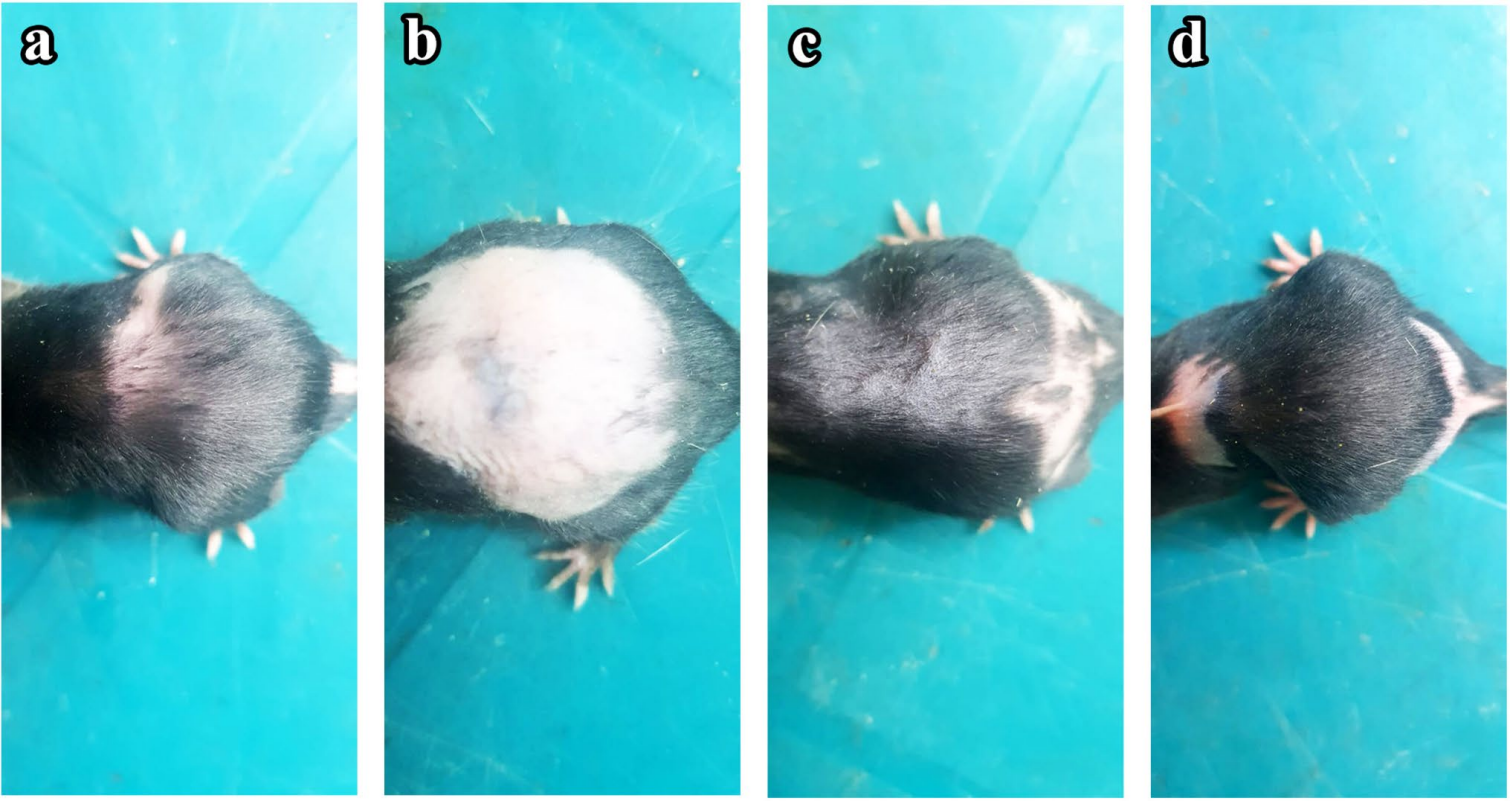
Photodocumentation of hair regrowth on the dorsal skin of the experimental groups at the end of the experiment (day 16). **a** Group I (control) C57BL/6 mice, **b** group II (CYP) C57BL/6 mice, **c** group III (CYP & NAC) C57BL/6 mice, and **d** group IV (NAC) C57BL/6 mice

On the 12th day after shaving, the hairs on the dorsal skin of C57BL/6 mice injected with CYP significantly fell off, which proved the success of the CIA mouse model. Hair growth promotion was evaluated by observing the darkening of the skin color which indicated telogen to anagen conversion (Fig. 1). Group I (control) mice (Fig. 1a) and group IV (NAC) mice (Fig. 1d) showed rapid growth of hair with almost full coat hair and blackened skin areas at the end of the experiment (day 16). Group  II (CYP) mice showed complete alopecia with no hair growth lasting until the day of sacrifice (day 16) (Fig. 1b). However, group III (CYP & NAC) mice manifested considerably rapid hair regrowth with black hair covering nearly the entire area of depilated skin at day 16 (Fig. 1c).

### Light microscopic results

#### H&E results

**Fig. 2:**
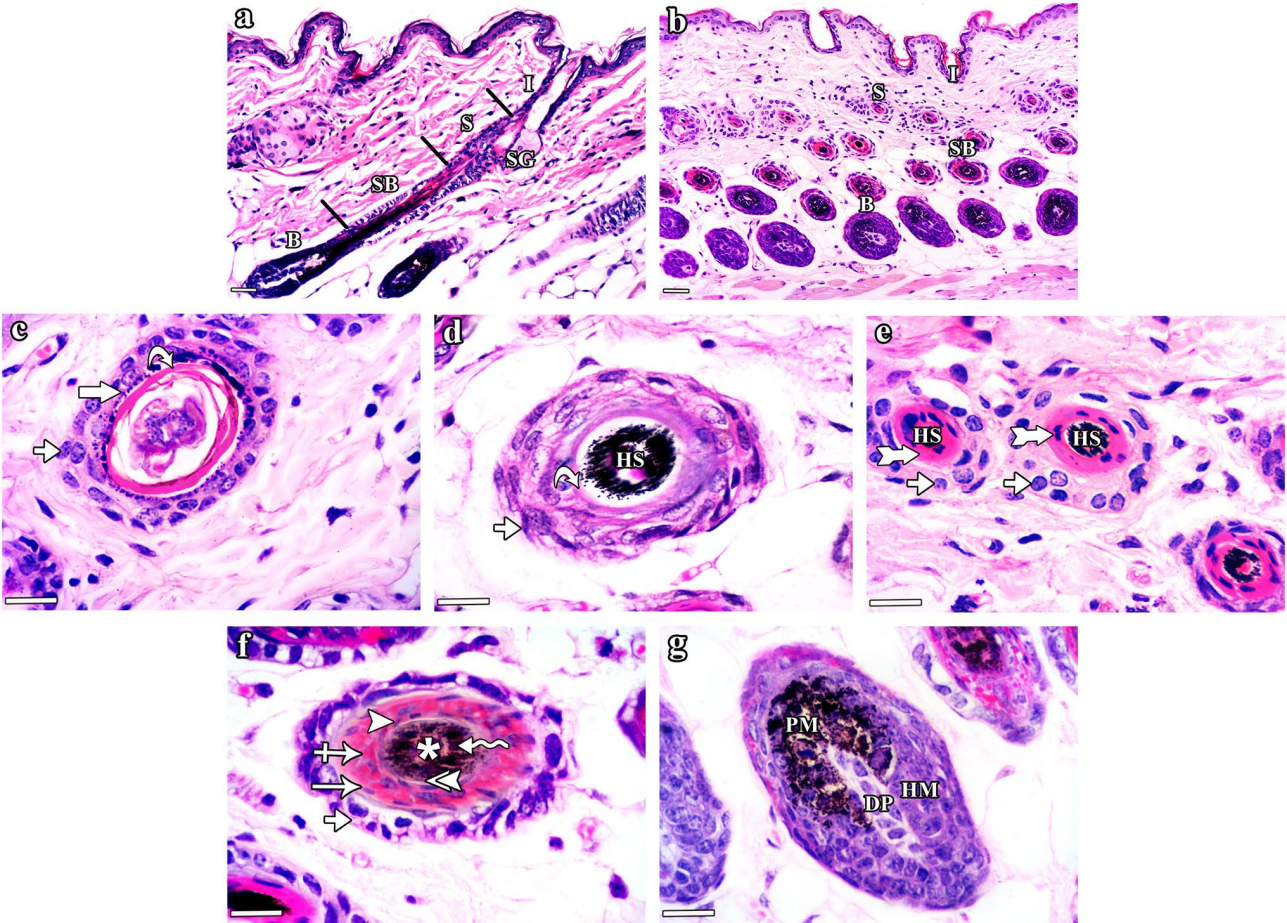
H&E stained sections of the dorsal skin of group I (control) C57BL/6 mice illustrating the normal histological structure of the different parts of the HF. **a **&** b** The HF is divided into: infundibulum (I), isthmus (S), supra-bulbar region (SB), and hair bulb (B). Note the opening of the sebaceous gland (SG) duct at the junction of the infundibulum (I) and isthmus (S). **c–g** Horizontal sections of the HF cut at different levels (from top to bottom). **c** The infundibulum is lined by the outer root sheath (ORS) formed of skin surface epidermis, which includes stratified squamous epithelium (short arrow) with keratohyaline granular layer (thick arrow) and basket-weave lamellar keratin (curved arrow). **d** The upper part of the isthmus is lined by ORS formed of stratified epithelium (short arrow) and compact homogeneous eosinophilic trichilemmal keratin (curved arrow). Note the presence of pigmented hair strand (HS) in the central part. **e** The lower part of the isthmus is lined by ORS and IRS. The ORS (short arrows) is formed of stratified epithelium and the IRS (tailed arrows) appears in the form of single fully cornified layer. Central hair strands (HS) are also seen. **f** The supra-bulbar level is formed of pale ORS and IRS. The ORS (short arrow) is formed of stratified epithelium with glycogenated keratinocytes and the IRS includes the Henle’s layer (arrow), Huxley’s layer with red trichohyaline granules (crossed arrow), and cuticle layer (arrowhead). A central hair formed of outer cuticle (double arrowhead), middle cortex (zigzag arrow), and inner medulla (asterisk) is also observed. **g** The bulbar level is formed of hair matrix cells (HM) and central spindle-like dermal papilla (DP). Pigmented melanocytes (PM) and melanin granules are present at the apex of the dermal papilla (DP). **a **&** b** Scale bars: 20 µm; **c–g** scale bars: 10 µm

H&E stained sections of group I (control) mice (Fig. 2) illustrated the normal histological structure of the HF composed of infundibulum, isthmus, supra-bulbar region, and hair bulb (Fig. 2a & b). At the level of the infundibulum, the HF was lined by the outer root sheath (ORS) having a degree of keratinization similar to that of the skin epidermis (stratified squamous epithelium with distinguishable keratohyaline granular layer and basket-weave lamellar keratin; Fig. 2c). The isthmus was lined by ORS formed of stratified epithelium exhibiting homogeneous eosinophilic trichilemmal keratin in its upper part and fully cornified single layer of inner root sheath (IRS) in its lower part (Fig. 2d & e). The supra-bulbar level showed ORS of stratified epithelium with glycogenated keratinocytes as well as IRS composed of three layers (Henle’s layer, Huxley’s layer, and cuticle layer). The Henle’s layer had a single layer of elongated cells, the Huxley’s layer exhibited three to four layers of cuboidals cells with red trichohyaline granules and the hair cuticle appeared as a single layer of overlapping elongated cells (Fig. 2f). The bulbar level embraced the spindle-shaped dermal papilla which was fully enclosed by matrix keratinocytes admixed with pigmented melanocytes at the apex of the dermal papilla (Fig. 2g). At the higher levels of the follicle, hair strands consisting of three layers (outer cuticle, middle cortex, and inner medulla) were visible.

**Fig. 3:**
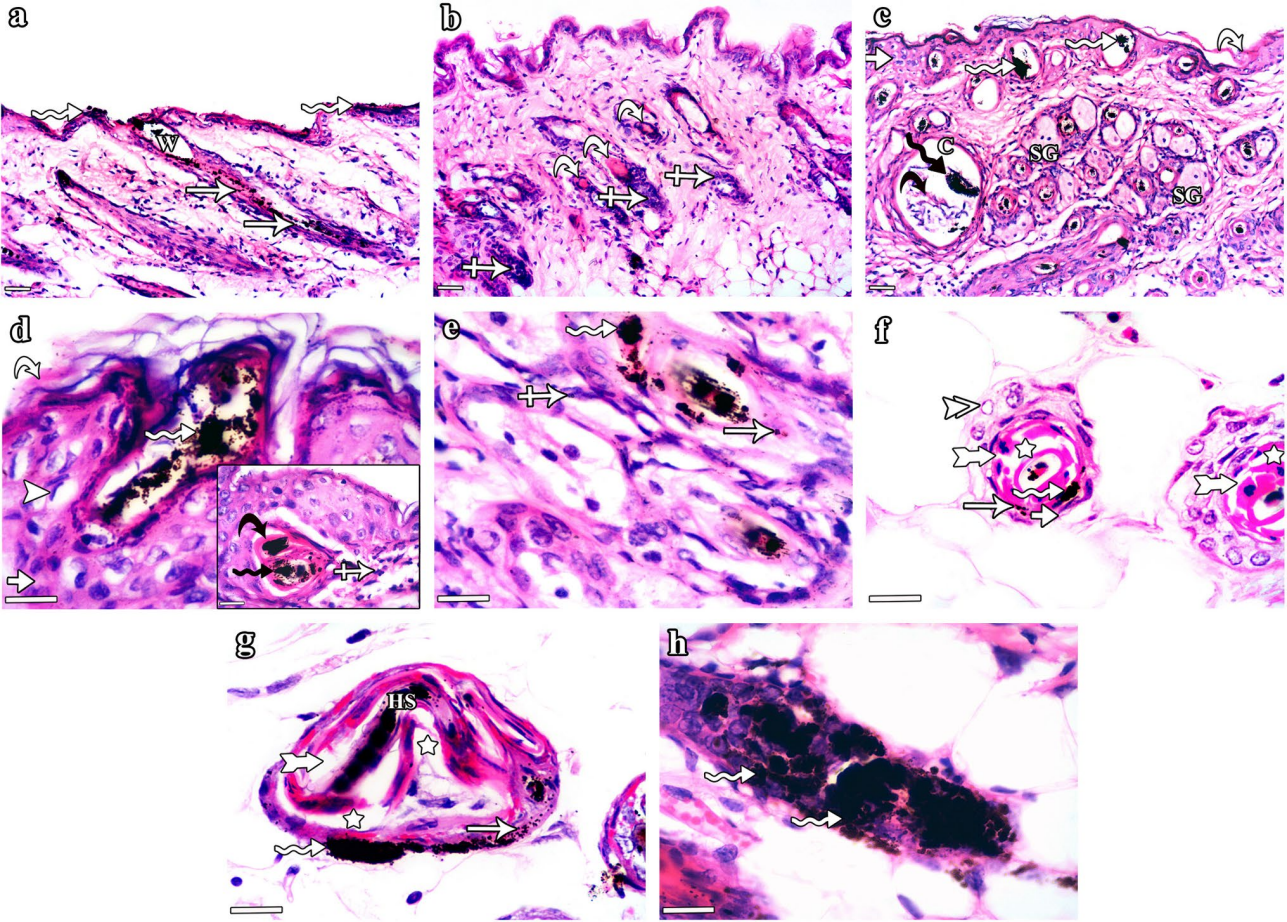
H&E stained sections of the dorsal skin of group II (CYP) C57BL/6 mice illustrating HF histological changes associated with CYP treatment. **a–c** HF dystrophy and disruption of follicular melanogenesis are seen in the form of: **a** widening of the hair canal (W) as well as abnormal intrafollicular (arrows) and epidermal (zigzag arrows) distribution of melanin granules, **b** follicular plugging by keratinaceous debris (curved arrows) and peri-follicular inflammatory cells (crossed arrows), and **c** HFs containing melanin clumps (zigzag arrows), enlarged sebaceous glands (SG), as well as HF-derived cysts (C) enclosing melanin clumps (black zigzag arrow) and keratinous material (black curved arrow). Hyperkeratosis (curved arrow) and hyperplasia (short arrow) of the interfollicular epidermis are also observed. **d–h** Sections of HFs cut at different levels (from top to bottom). **d** The infundibulum exhibits hyperplasia (short arrow) of the epithelial lining with some vacuolated cells having nuclear margination (arrowhead), hyperkeratosis (curved arrow), and aggregation of melanin (zigzag arrow). Inset: keratinous bleb (black curved arrow) containing melanin clumps (black zigzag arrow) and perifollicular inflammatory cells (crossed arrow) are seen. **e** The upper part of the isthmus appears distorted with ectopic melanin granules (arrow), melanin clumps (zigzag arrow), and surrounding inflammatory cells (crossed arrow). **f** The lower part of the isthmus shows ORS with eccentric thinning of the epithelium (short arrow) and some cells having ring-shaped marginal chromatin (double arrowhead). The IRS is distorted (tailed arrows) with slight separation (stars) and abnormally located melanin granules (arrow) and clumps (zigzag arrow). **g** The supra-bulbar level shows a twisted irregular appearance. The ORS illustrates ectopic distribution (arrow) and clumping (zigzag arrow) of melanin, while the IRS appears disorganized (tailed arrow) with abnormal cell–cell spaces between layers (stars) and central kinked hair stand (HS). **h** The bulbar level has an irregular shrunken diameter with the presence of melanin clumps (zigzag arrows). **a–c** Scale bars: 20 µm; **d–h** scale bars: 10 µm

Group II (CYP) mice illustrated different aspects of HF dystrophy and disruption of follicular melanogenesis (Fig. 3). Distortion of the HFs was observed in the form of widening of the hair canals, ectopic melanin granules, clumping of melanin, follicular plugging by keratinaceous debris and perifollicular inflammatory cells (Fig. 3a, b & c). In the infundibulum, hyperplasia of the epidermis with vacuolated cells having nuclear margination, hyperkeratosis, keratinous blebs containing melanin clumps were also seen (Fig. 3d). The isthmus appeared distorted with marginal chromatin arrangement in cells lining the ORS and degenerated IRS (Fig. 3e & f). The supra-bulbar level appeared irregularly twisted with abnormal cell–cell spaces between layers, disorganized IRS and distorted kinked hair strands (Fig. 3g). The bulbar level was shrunken with irregular diameters of the hair bulbs (Fig. 3h).

**Fig. 4:**
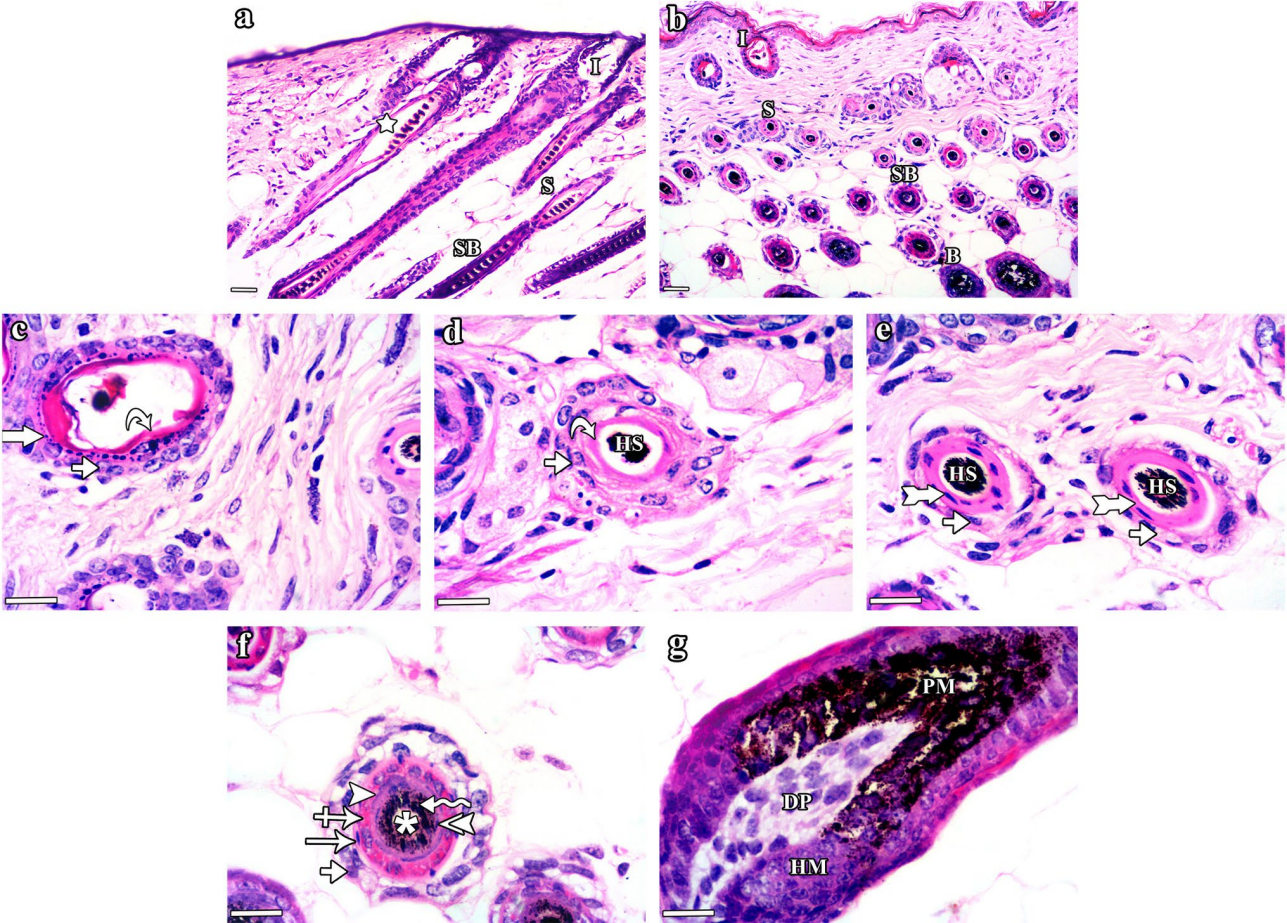
H&E stained sections of the dorsal skin of group III (CYP & NAC) C57BL/6 mice illustrating lower degree of HF dystrophy and preserved follicular architecture almost similar to that in the control group. **a **&** b** The HF is divided into: infundibulum (I), isthmus (S), supra-bulbar region (SB), and hair bulb (B). However, slight separation between the central hair and the surrounding HF is seen in certain sites (star). **c–g** Horizontal sections of the HF cut at different levels (from top to bottom). **c** The infundibulum is lined by ORS formed of stratified squamous epithelium (short arrow) of the skin epidermis with its characteristic keratohyaline granular layer (thick arrow) and lamellar keratin (curved arrow). **d** The upper part of the isthmus is lined by ORS formed of stratified epithelium (short arrow) and homogeneous trichilemmal keratin (curved arrow). Central pigmented hair strand (HS) is also seen. **e** The lower part of the isthmus is formed of stratified epithelium of the ORS (short arrows) and fully cornified IRS (tailed arrows) surrounding the hair strands (HS). **f** The supra-bulbar level is formed of highly preserved ORS and IRS. The ORS is formed of stratified epithelium (short arrow) and the IRS consists of Henle’s layer (arrow), Huxley’s layer with red trichohyaline granules (crossed arrow), and cuticle layer (arrowhead). Central hair formed of cuticle (double arrowhead), cortex (zigzag arrow), and the innermost medulla (asterisk) is also observed. **g** The bulbar level is formed of hair matrical cells (HM), pigmented dendritic melanocytes (PM), and dermal papilla (DP). **a **&** b** Scale bars: 20 µm; **c–g** scale bars: 10 µm

**Fig. 5:**
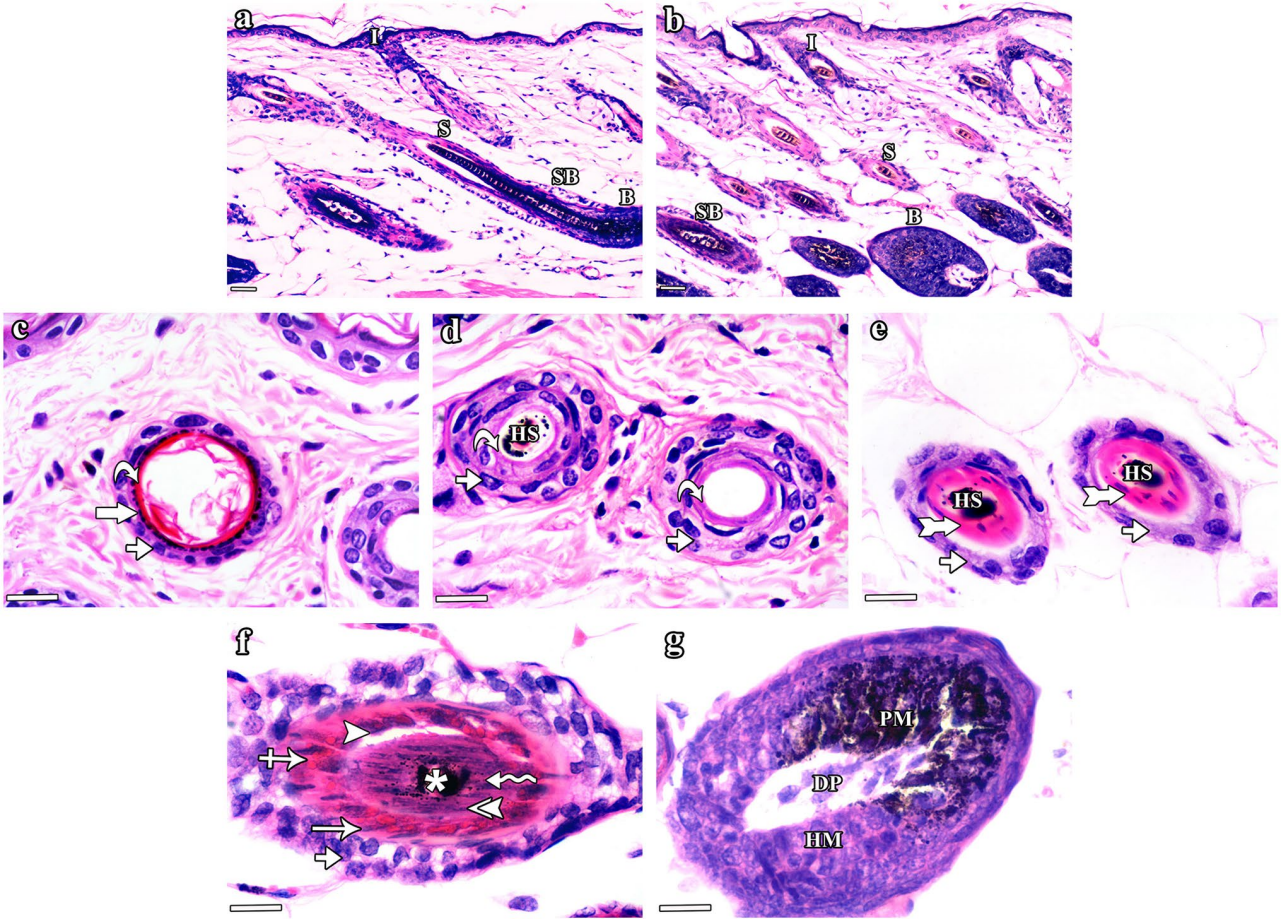
H&E stained sections of the dorsal skin of group IV (NAC) C57BL/6 mice illustrating the normal histological structure of the different parts of the HFs as appeared in the control group. **a **&** b** The HF is divided into: infundibulum (I), isthmus (S), supra-bulbar region (SB), and hair bulb (B). **c–g** Horizontal sections of the HF cut at different levels (from top to bottom). **c** The infundibulum is lined by ORS formed of the thin skin surface epidermis: stratified squamous epithelium (short arrow) with granular layer (thick arrow) and lamellar keratin (curved arrow). **d** The upper part of the isthmus is lined by stratified epithelium (short arrows) of the ORS with eosinophilic trichilemmal keratin (curved arrows) surrounding the hair strand (HS). **e** The lower part of the isthmus is lined by ORS (short arrows) and fully cornified IRS (tailed arrows). Pigmented hair strands (HS) are also seen. **f** The supra-bulbar level is formed of pale ORS and IRS. The ORS is formed of stratified epithelium (short arrow) and the IRS includes the Henle’s layer (arrow), Huxley’s layer (crossed arrow), and cuticle layer (arrowhead). The central hair is formed of cuticle (double arrowhead), cortex (zigzag arrow), and medulla (asterisk). **g** The bulbar level is formed of hair matrix cells (HM), central spindle-like dermal papilla (DP), and pigmented melanocytes (PM). **a **&** b** Scale bars: 20 µm; **c**–**g** scale bars: 10 µm

Group III (CYP & NAC) mice showed a lower degree of HF dystrophy and preserved follicular architecture almost similar to that in the control group (Fig. 4). HFs divided into well-organized infundibulum, isthmus, supra-bulbar region, and hair bulb were seen (Fig. 4a–g). However, slight separation between the central hair and the surrounding HF was still seen in certain sites (Fig. 4a). Group IV (NAC) mice (Fig. 5) showed the normal structure of the HF (Fig. 5a & b) composed of infundibulum (Fig. 5c), isthmus (Fig. 5d & e), supra-bulbar region (Fig. 5f), and hair bulb (Fig. 5g).

### Schmorl’s stain results

**Fig. 6:**
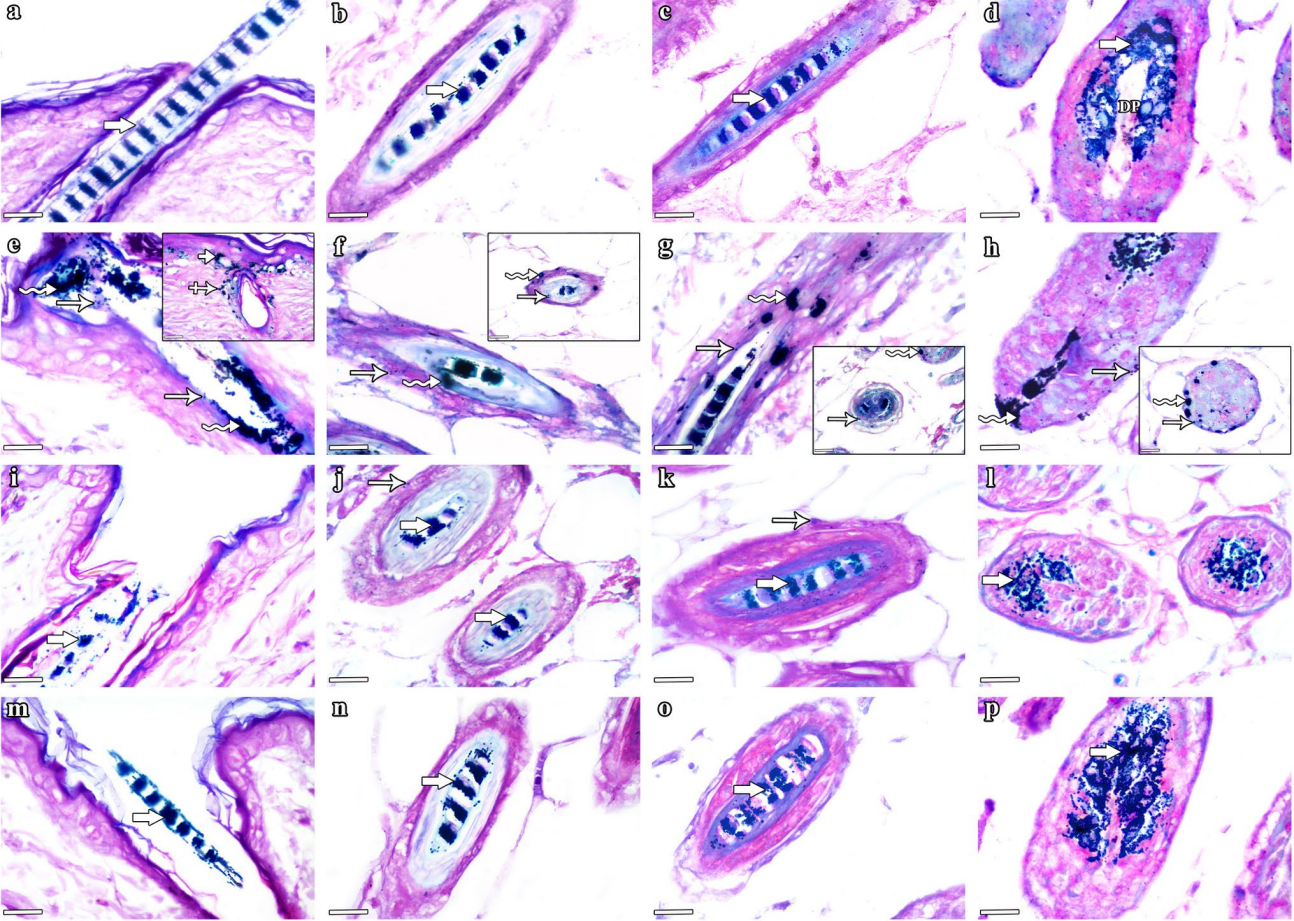
Schmorl’s stained sections of HFs cut at different levels illustrating the pattern of follicular melanin distribution in the experimental groups. Group I (control) mice show hair strands with typical intrafollicular zebra-stripe pattern of melanin distribution (thick arrows) in: **a** the infundibulum, **b** the isthmus, and **c** the supra-bulbar level. In **d**, the hair bulb exhibits the characteristic inverted Y-shaped melanin granules (thick arrow) around the dermal papilla (DP). Group II (CYP) mice reveal disturbances of follicular melanogenesis in the form of ectopic melanin distribution (arrows) and melanin clumping (zigzag arrows) in: **e** the infundibulum, **f** the isthmus, **g** the supra-bulbar level, and **h** the hair bulb. Note the presence of abnormal ectopic perifollicular (crossed arrow) and epidermal interfollicular (short arrow) melanin accumulation in certain sites **(Inset in e)**. Group III (CYP & NAC) mice show preservation of normal melanin localization (thick arrows) in: **i** the infundibulum, **j** the isthmus, **k** the supra-bulbar level, and **l** the hair bulb. Occasional small ectopic melanin granules (arrows) are still seen in few sites in **j **&** k**. Group IV (NAC) mice exhibit the characteristic pattern of normal melanin distribution (thick arrows) in: **m** the infundibulum, **n** the isthmus, **o** the supra-bulbar level, and **p** the hair bulb. **a–p** Scale bars: 10 µm

Schmorl’s stained sections of group I (control) mice (Fig. 6a–d) and group IV (NAC) mice (Fig. 6m–p) illustrated the normal intrafollicular melanin distribution. Hair strands in the infundibulum, isthmus, and supra-bulbar levels showed the zebra-stripe pattern of melanin arrangement, while the hair bulb revealed the characteristic inverted Y-shaped melanin granules around the dermal papilla. Group II (CYP) mice revealed disturbances of follicular melanogenesis in the form of ectopic melanin distribution and melanin clumping in the different levels of HFs as well as the presence of abnormal ectopic perifollicular and epidermal interfollicular melanin localization (Fig. 6e–h). Group III (CYP & NAC) mice showed preservation of the normal characteristic melanin distribution (Fig. 6i–l) with the presence of occasional small ectopic melanin granules in few sites (j & k).

### Immunohistochemical results and statistical analysis

#### Anti-p53 results

**Fig. 7:**
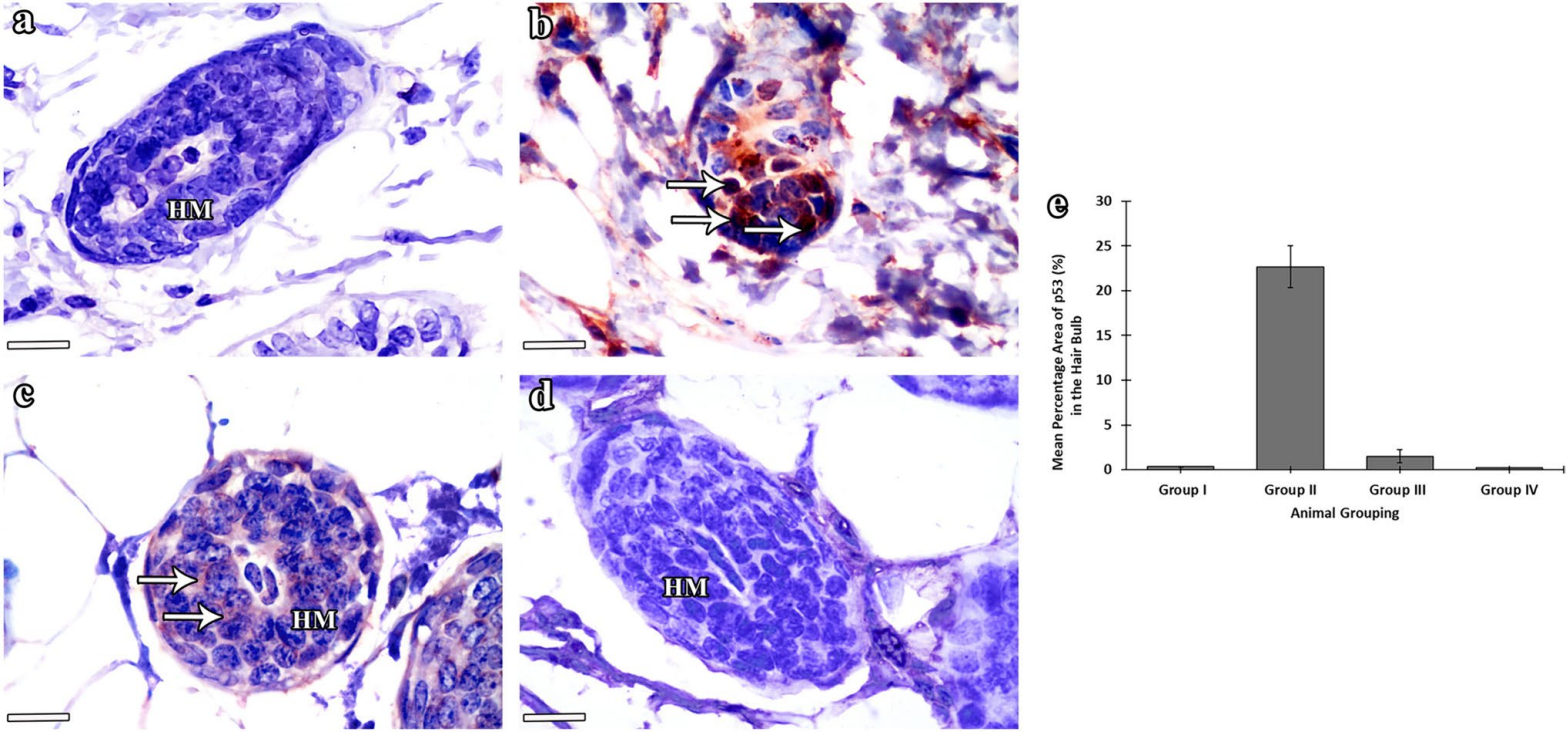
Anti-p53 expression among the experimental groups. **a–d** Anti-p53 stained sections of the HF bulb illustrating the differences in p53 immunoreactivity among the experimental groups. **a–d** Scale bars: 10 µm. **a** Group I (control) mice show negative immune reaction for p53 in the hair matrix cells (HM). **b** Group II (CYP) mice reveal positive p53 reaction (arrows) in the majority of hair matrix cells. **c** Group III (CYP & NAC) mice show the majority of hair matrix cells expressing negative immune reaction for p53 (HM) with the presence of occasional cells having faint cytoplasmic reaction (arrows). **d** Group IV (NAC) mice show negative p53 immune expression in the hair matrix cells (HM). **e** Statistical study of the percentage area (%) of p53 (mean ± SD) in the follicular hair bulb of the studied groups

Examination of anti-p53 stained sections of group I (control) mice (Fig. 7a) and group IV (NAC) mice (Fig. 7d) showed negative p53 immune expression in the hair matrix cells with statistically non-significant differences (*P* > 0.05) between both groups. Group II (CYP) mice showed hair matrix cells expressing positive immunoreactivity for p53 reaction (Fig. 7b) evidenced by the statistically highly significant increase (*P* < 0.001) in the percentage area of p53 in the hair bulb as compared with that of the control group I. Group III (CYP & NAC) mice showed the majority of hair matrix keratinocytes with negative reactivity for p53 (Fig. 7c) with statistically high significant decrease in p53 percentage area compared with CYP group II (*P* < 0.001) and significant increase as compared with control group I (*P* < 0.05).

### Anti-ki67 results

**Fig. 8:**
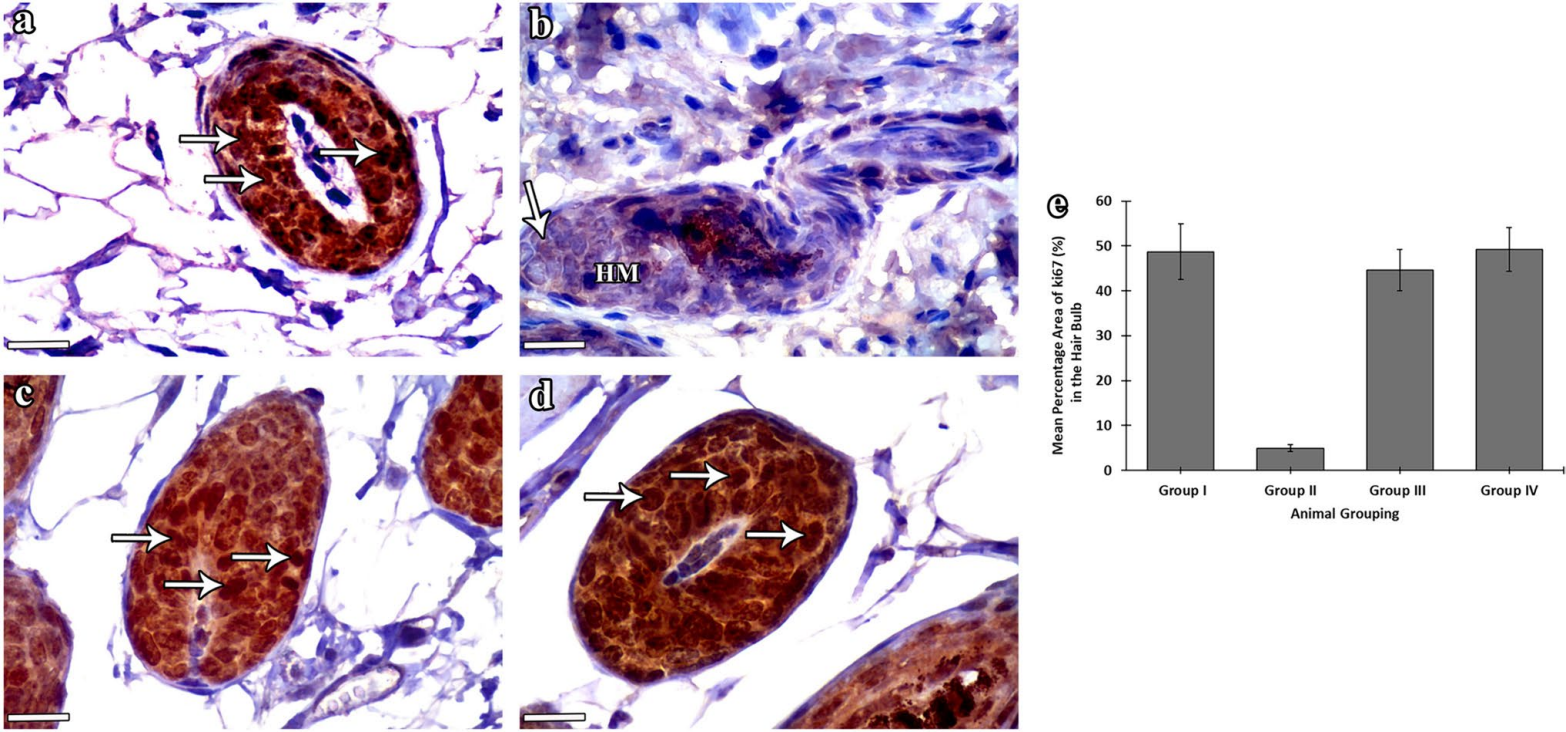
Anti-ki67 expression among the experimental groups. **a–d** Anti-ki67 stained sections of the HF bulb. **a–d** Scale bars: 10 µm. **a** Group I (control) mice show a strong brown positive ki67 immune reaction in the hair matrix cells (arrows). **b** Group II (CYP) mice exhibit almost negative ki67 reaction in the hair matrix (HM). A faint reaction may be seen in few matrix cell nuclei (arrow). **c** Group III (CYP & NAC) mice show strong positive reaction in the majority of the matrix cells (arrows). **d** Group IV (NAC) mice show a strong Ki67 immunostaining pattern in the hair matrix cells (arrows). **e** Statistical study of the percentage area (%) of ki67 (mean ± SD) in the follicular hair bulb of the studied groups

The hair matrix cells of group I (control) mice (Fig. 8a) and group IV (NAC) mice (Fig. 8d) showed a strong positive ki67 immune reaction, with statistically nonsignificant differences (*P* > 0.05) between both groups. Group II (CYP) mice showed hair matrix cells expressing negative immune reaction for ki67 (Fig. 8b). This finding was statistically confirmed by the high significant decrease (*P* < 0.001) in ki67 percentage area in CYP mice as compared with those of control ones. Group III (CYP & NAC) mice showed the majority of hair matrix keratinocytes expressing positive reaction for ki67 (Fig. 8c), which was statistically represented by the high significant increase (*P* < 0.001) in the percentage area of ki67 compared with the CYP group (group II) and nonsignificant decrease as compared with control group I (*P* > 0.05).

### Biochemical results and statistical analysis

#### MDA

**Fig. 9:**
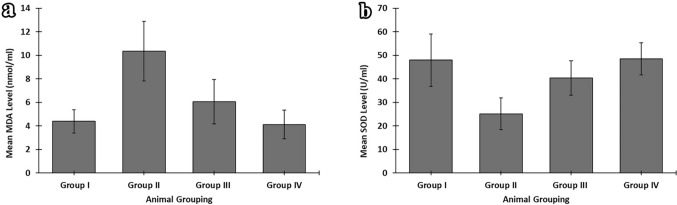
Statistical study of the level of oxidative stress markers (mean ± SD) in the experimental groups. **a** Mean (± SD) level of MDA (nmol/ml). **b** Mean (± SD) level of SOD (U/ml)

Serum level of MDA of group I (control) mice and group IV (NAC) mice showed nonsignificant differences with each other (*P* > 0.05). Group II (CYP) mice showed a highly significant increase in the serum level of MDA compared with that of the control group (group I) (*P* < 0.001). Group III (CYP & NAC) mice showed high significant decrease as compared with group II (CYP) mice (*P* < 0.001) and significant increase as compared with the control group (group I) (*P* < 0.05) (Fig. 9a).

#### SOD

Serum level of SOD of group I (control) mice and group IV (NAC) mice showed nonsignificant differences with each other (*P* > 0.05). Group II (CYP) mice showed a highly significant decrease in the serum level of SOD as compared with that of group I (*P* < 0.001). Group III (CYP & NAC) mice showed high significant increase as compared with group II (CYP) mice (*P* < 0.001) and significant decrease as compared with the control group (group I) (*P* < 0.05) (Fig. 9b).

## Discussion

Anagen HFs have high mitotic activity of hair matrix cells, and that makes them vulnerable to chemotherapeutic agents (Kim et al. 2021). Moreover, the melanocytes of the HF pigmentary unit, generating enormous amounts of melanin in anagen, are also targets of chemotherapy (Yoon et al. 2016). Effect of chemotherapy on anagen HFs and their pigmentary units and the subsequent hair loss cause not only psychosocial stress in cancer patients but also chemotherapy refusal. Unfortunately, absence of the exact pathophysiological mechanisms of CIA has made it difficult to discover efficient preventive or therapeutic procedures for this side effect (Kim et al. 2019).

The present study showed that CYP administration causes hair loss, histological dystrophic changes in the HFs, as well as deterioration of the biochemical parameters in adult female C57BL/6 mice. Similar findings of CYP-induced hair loss and alopecia in mice were also reported by Chen et al. (2016) and could be attributed to the massive apoptosis in the maximally proliferating hair matrix cells (Haslam et al. 2021). The HF dystrophic changes in H&E stained sections of the CYP group coincide with the earlier reports by Onaolapo et al. (2018). Studies have shown that chemotherapeutic agents affect hair matrix cells by decreasing the growth factors, including epidermal growth factor, keratinocyte growth factor, transforming growth factor, and parathyroid hormone-related protein, resulting in loss of HF ability to proliferate (Kim et al. 2021).

CYP can induce toxicity via various mechanisms. It activates mitogen-activated protein kinase (MAPK)/nuclear factor kappa-B (NF-κB) signaling pathways with subsequent elevation in the gene expression of numerous inflammatory cytokines as interleukin (IL)-1β, IL-6, and tumor necrosis factor-α (TNF-α) (Zhu et al. 2022). In addition, CYP administration results in damage of DNA, production of ROS, and stimulation of mitochondrial and endoplasmic reticulum (ER) stresses. Excess proinflammatory cytokines and ROS work together to induce cell death via apoptosis (Al-Amarat et al. 2022). Cells with nuclear margination and those with ring-shaped chromatin condensation in CYP-treated mice of the present study are signs of cell death and apoptosis associated with toxic agents (Creasy and Chapin 2018; Singh and Singh 2019).

The disturbances of follicular melanogenesis with CYP administration in group II mice were also reported by Böhm et al. (2014) and Haslam et al. (2021). Melanocytes are unintended targets for chemotherapeutic agents. CYP administration alters the expression of proteins involved in melanogenesis, apoptosis, proliferation, and migration of follicular melanocytes of C57BL/6 mice. CYP induces apoptosis of some melanocytes in the hair bulb region through the Fas signaling pathway. The remaining surviving hair bulb melanocytes express c-kit receptor which causes proliferation and migration of follicular melanocytes up the ORS of the HF, and later their subsequent localization in the inter-follicular epidermis. Fas and c-kit signaling pathways are the reasons for skin hyperpigmentation that occurs as an adverse effect of chemotherapy (Sharov et al. 2003).

The present study findings of increased the percentage area of p53 and decreased that of ki67 in the HF matrix cells of CYP-treated mice are supported by the previous results by Yoon et al. (2016) and Kim et al. (2020), respectively. DNA damage in the HFs by chemotherapy leads to accumulation of p53 tumor suppressor protein with subsequent up-regulation of Fas, insulin-like growth factor binding protein (IGFBP)-3 and Bcl-2-associated x (Bax) encoded by the corresponding p53-responsive genes resulting in HF apoptosis (Chen et al. 2016). Ki67 is a nuclear antigen present in all phases of cell cycle and it is the most commonly used cell proliferation marker (Andrés–Sánchez et al. 2022). In addition to apoptosis, loss of hair matrix cell proliferation expressed by almost negative ki67 immunoreactivity is a main mechanism through which chemotherapy can induce damage in the HFs (Azhagu Saravana Babu et al. 2023).

CYP-induced increase in the level of MDA with accompanying decrease in that of SOD was also reported by Chen et al. (2022) and indicates a role of oxidative stress in the HF damage observed with CYP treatment. CYP is metabolized in the liver by cytochrome-P450 mixed function oxidase enzymes to produce oxidative agents causing excess generation of free radicals. The free radical formation stimulates oxidative damage of various cell components, membrane lipid peroxidation and defects in enzymes, including antioxidant enzymes. The elevation of ROS induced by CYP enhances the production of proinflammatory mediators (Althunibat et al. 2023) and stimulates apoptosis through the expression of various proapoptotic proteins, such as Bax and caspases (Ullrich et al. 2022). Therefore, suppression of oxidative stress and consequent attenuation of proinflammatory and cell death pathways can be of significant therapeutic benefit.

Simultaneous administration of NAC with CYP in group III showed a significant improvement of the biochemical deterioration as well as the HF degenerative histological changes induced by CYP. Similar results of the beneficial effects of NAC in improving alopecia have been also reported in androgenic alopecia in men (El Sayed et al. 2021) and scarring alopecia in patients with lichen planopilaris (Ahmadi Kahjoogh et al. 2022).

The significant preservation of the histological architecture of the HF in H&E and Schmorl’s stained sections in group III (CYP & NAC) mice may be attributed to the antiinflammatory effect of NAC, which could be owing to its ability to inhibit the activation of the proinflammatory NF-κB (Smaga et al. 2021) and reduce ROS (Mardani et al. 2021). Preservation of normal melanin distribution in the current study could be explained by the antioxidant properties of NAC that enhances the survival of melanocyte stem cells that differentiate into melanocytes (Wilson et al. 2023) as melanocytes are vulnerable to oxidative stress owing to their reduced antioxidant capability compared with other cells present in the skin (Enkhtaivan & Lee 2021).

The decrease in the percentage area of p53 induced by NAC in group III (CYP & NAC) mice is in agreement with the previous results of NAC-induced p53 down regulation in cisplatin-induced hepatotoxicity (Coşkun et al. 2021) and burn-induced skin injuries (Sadaqat et al. 2022). The antiapoptotic mechanism of NAC may include down regulating p53 and Bax levels and increasing the Bcl-2 mRNA expression (Mantawy et al. 2020). NAC effect might be mediated via the p38- MAPK signaling pathway, which has a critical role in cell apoptosis (Ma et al. 2022; Abusaliya et al. 2023) as well as an effect on decreasing ROS (Abedini Bajgiran et al. 2023).

In the present study, the NAC-induced increase in the expression of ki67 in group III (CYP & NAC) mice could be explained by renormalization of keratinocyte proliferation and differentiation with subsequent formation of a regular cornified envelope (Parasassi et al. 2014). Janeczek et al. (2019) reported that NAC engagement in decreasing free radicals and increasing GSH concentrations helps in epidermal proliferation, therefore, it may be useful for dermatological problems. Moreover, NAC increases cell migration, scratch wound healing activities, and epithelization-related proteins in the skin.

The improvement in oxidative stress markers (MDA & SOD) associated with NAC administration in the current work comes in agreement with Satvati et al. (2022). NAC is a precursor of the main antioxidant system GSH. Furthermore, a relevant mechanism in the antioxidant activity of NAC is owing to the scavenging of ROS and reactive nitrogen species (Oliva et al. 2023). In addition, NAC improves mitochondrial function and promotes the nuclear translocation of nuclear factor erythroid 2‐related factor 2 that enhances the expression of the antioxidant enzymes SOD and GSH peroxidase (Fan et al. 2022).

## Conclusions

NAC administration reversed CYP-induced alterations in the biochemical parameters and preserved HF structure, typical follicular melanin distribution, and the normal pattern of p53 and ki67 expression. These findings confirmed the reported beneficial effects of NAC on CIA and indicated that NAC could be used as an efficient and safe therapeutic option for hair loss induced by chemotherapy.

## Data Availability

No datasets were generated or analysed during the current study.
